# Quantitative detection of methylated NDRG4 gene as a candidate biomarker for diagnosis of colorectal cancer

**DOI:** 10.3892/ol.2014.2815

**Published:** 2014-12-19

**Authors:** WENHUA XIAO, HUIXIA ZHAO, WEIWEI DONG, QUIWEN LI, JIANHUA ZHU, GUANGHUI LI, SHUFANG ZHANG, MING YE

**Affiliations:** Department of Oncology, The First Affiliated Hospital of Chinese PLA General Hospital, Beijing 100048, P.R. China

**Keywords:** NDRG4 methylation, colorectal cancer, methylation-specific PCR, blood, stools, urine

## Abstract

The present study explored the use of methylated NDRG4 gene as a candidate biomarker for diagnosis of colorectal cancer (CRC). Methylated NDRG4 gene expression from colorectal carcinoma tissue, paracarcinoma tissues, stools, blood and urine were detected successfully in DNA samples from 84 patients, by nested methylation-specific polymerase chain reaction and denaturing high-performance liquid chromatography. The sensitivity and specificity of methylated NDRG4 gene expression for us as a biomarker in colorectal cancer was analyzed and compared with 16 age-matched healthy controls. The positive detection rate of methylated NDRG4 was 81% in carcinoma tissue, 8.3% in paracarcinoma tissues, 54.8% in blood, 72.6% in urine and 76.2% in stools. Considering the convenience of the acquisition of urine samples, an additional group of 76 patients with CRC were recruited for verification of detecting methylated NDRG4 in the urine. The positive detection rate of methylated NDRG4 was 72.4% (55/76) in this cohort. The detection of methylated NDRG4 in stools and urine could be used as a novel diagnostic technique for highly sensitive and specific detection of CRC. Due to the ease of collecting urine samples, this novel method could be a potential biomarker for early diagnosis of CRC.

## Introduction

Colorectal cancer (CRC) is the second leading cause of cancer-related mortality and the third most prevalent cancer in western countries (1–3). CRC is curable in its early stages, whereas in the advanced stages, the survival time is <30 months. Early diagnosis and treatment is therefore of high importance (4). The current routine method of screening is by colonoscopy. The use of colonoscopy, however, is not an ideal method since it is an invasive technique and the use of colonoscopy screening could be limited in high-risk populations, in particular in low-income patients (5,6). The fecal occult blood test for CRC is an additional screening method that is currently performed, however this test is limited due to its low sensitivity (7). Studies have therefore been performed to improve the diagnostic biomarkers available for detection of CRC (8,9).

Abnormal DNA methylation is an early event that occurs during tumorigenesis, and numerous abnormally hypermethylated genes have been identified in cases of CRC (10–12). Gene methylation status could therefore be used as a molecular diagnostic indicator (13). DNA from CRC cells can be readily obtained from feces samples, which contain both normal and CRC-shedded colorectal epithelial cells, from which DNA can be extracted (14). In addition, the alkaline environment of the intestine is conducive to the preservation of DNA (15). NDRG4 is a member of the NDRG gene family and is a known tumor suppressor gene (16,17). A previous study suggested that the NDRG4 gene is present in CRC tumor tissues and bodily fluids with high stability and repeatability (16).

The present study analyzed the methylated NDRG4 gene expression in 87 patients with CRC by nested methylation-specific polymerase chain reaction (n-MSP) combined with denaturing high-performance liquid chromatography (DHPLC). The samples were collected from carcinoma and paracarcinoma tissues, blood, urine and feces of patients with CRC. The sensitivity and specificity of methylated NDRG4 gene detection in these samples were compared with those in healthy controls, and assessed for use as a potential candidate biomarker for the diagnosis of CRC.

## Materials and methods

### Patient enrollment

The present study was approved by the Institutional Review Boards of the General Hospital of PLA (Beijing, China). The CRC and pericarcinous tissues were obtained from surgeries undertaken between June 2010 and August 2011. All the samples were confirmed by pathology. All patients provided written informed consent, and the patients did not receive chemotherapy or radiotherapy before the surgical procedure. Stool and urine samples were collected prior to the surgery, from which genomic DNA was immediately extracted and then stored at −20°C. Cancer and pericarcinous tissue specimens were stored at −80°C for further use.

Following screening, a total of 87 CRC patients were recruited in the study, of which 56 cases were colon cancers and 31 cases were rectal cancers. In the study group, 52 were males and 35 were females, with an age range between 38 and 73 years, and a median age of 56. A cohort of 16 age-matched healthy subjects were recruited as a control, of which 9 were males and 6 were females, with an age range between 40 and 74 years, with a median age of 55.6.

### DNA isolation

Homogenate-tissue DNA was extracted using the TGuide Tissue Genomic DNA Extraction kit [Tiangen Biotech (Beijing) Co., Ltd, Beijing, China]. DNA from fecal samples was extracted using a Stool DNA Extraction kit (Bioneer Corporation, Daedeok-gu, Republic of Korea). Genomic DNA was extracted from total blood, using a TGuide Blood Genomic DNA Extraction kit [Tiangen Biotech (Beijing) Co., Ltd.], according to the manufacturer’s instructions.

### Bisulfite modification of genomic DNA

Following bisulfite modification, the unmethylated cytosine in genomic DNA will be converted to uracil, whereas methylated cytosine will not be converted. Tissue genomic DNA methylation was performed using a Wizard DNA Clean-up system (Promega Corporation, Madison, WI, USA) according to the manufacturer’s instructions. Stool and blood genomic DNA methylation were performed using an EZ DNA Methylation TM-Direct kit (Zymo Research Corporation, Irvine, CA, USA), according to the manufacturer’s instructions.

### n-MSP

The n-MSP was performed to identify the expression of NDRG4 genes from blood, urine, stool and tissue. The primer sequences and polymerase chain reaction (PCR) conditions for the n-MSP approach have been previously described (18,19). The primer sequences, annealing temperatures, and PCR sizes are listed in Table I. Methylated DNA from normal human lymphocytes, which was treated by *Sss*I methylase (New England BioLabs, Ipswich, MA, USA) was prepared as the positive control for methylated (M) amplifications, and DNA from untreated lymphocytes served as a negative control for unmethylated (U) amplifications.

The n-MSP of NDRG4 was performed in a 25-μl reaction volume, with 0.5 μl Taq polymerase (Invitrogen Life Technologies, Carlsbad, CA, USA) and 2 μl DNA template. The NDRG4 gene cycling conditions for the first round were as follows: 95°C for 15 min; 25 cycles of 95°C for 30 sec, 56°C for 30 sec, and 72°C for 30 sec; final extension at 72°C for 7 min. The cycling conditions for the second round were as follows: Preheating at 95°C for 15 min, 35 cycles of 95°C for 30 sec, 66°C for 30 sec, and 72°C for 30 sec; final extension at 72°C for 7 min. MSP products were analyzed by 2% polyacrylamide gel electrophoresis, and a product size of100 bp was expected for the NDRG4 gene.

### Statistical analysis

A χ^2^ test was used for methylation detection rate comparison between each sample of NDRG4. P<0.05 was considered to indicate a statistically significant difference. SPSS 13.0 software was used for all statistical analyses (SPSS, Inc., Chicago, IL, USA).

## Results

### Rate of NDRG4 methylation

**Figure 1:**
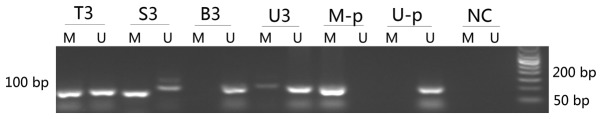
Nested methylation-specific polymerase chain reaction detection of methylated NDRG4 gene in carcinoma tissues, feces, urine and blood of a representative colorectal cancer case (patient no. 3). T, carcinoma tissues; S, stool; B, blood; U, urine; M, methylated products (88 bp); U, unmethylated products (100 bp); 3, patient no. 3; M-p, positive control of methylated DNA; U-p, positive control of unmethylated products; NC, negative control; bp, base pairs.

nMSP detection was successfully used to assess for methylated NDRG4 in carcinoma and paracarcinoma tissues, feces, urine and blood of the 84 CRC cases (Fig. 1). The positive rate of methylated NDRG4 gene expression was 81% (68/84) in carcinoma tissues, while the positive rate was only 8.3% (7/84) in paracarcinoma tissues. There was a significant difference in the levels of methylated NDRG4 in carcinoma as compared with the paracarcinoma tissues (P<0.01). The sensitivity and specificity of NDRG4 gene detection in the diagnosis of CRC was 81 (68/84) and 91.7% (77/84) in tissues, respectively.

In addition to analysis in the tissues, the sensitivity and specificity of methylated NDRG4 in feces, urine and blood were 54.8 and 78.1% in blood; 72.6 and 85% in urine; 76.2 and 89.1% in feces. Methylated NDRG 4 genes in feces and urine showed a higher sensitivity and specificity for diagnosis than blood. From the present data, it could be suggested that a combination of using urine and fecal samples to detect the methylation status of NDRG4 could be a beneficial diagnostic method for CRC.

### Highly expressed methylated NDRG 4 gene in tissue and feces DNA of CRC patients

The association between methylated NDRG4 and clinical pathological parameters were analyzed. The data showed no correlation between all methylated NDRG4 with age, the degree of tumor differentiation and TNM stage. Methylated NDRG4 in metastasis-lymph node tumors from CRC tissues and feces was significantly highly expressed as compared with non-metastatic samples (P<0.05). Methylated NDRG4 was not expressed highly in urine or blood samples (Table II).

### Early diagnosis of colorectal cancer by methylated NDRG 4 gene in urine

According to the above research results, we recruited another 76 CRC patients; they all were confirmed by pathology diagnosis, the median age of them was 54 years (range, 39–71), and 36 non-cancer patients were selected as the normal control group. Urine specimens were collected preoperatively in all patients, with a collection volume of 50–100 ml, then sent to the laboratory immediately, where genomic DNA were extracted and then frozen at −20°C. All patients did not receive preoperative chemotherapy or radiotherapy, and informed consent was obtained. Of the 76 CRC patients, the proportion of positive methylated NDRG 4 gene status was 72.4% (55/76). This result showed consistency with the former experiments; combining the convenience and sensitivity, methylated NDRG 4 gene testing in urine could be a potential testing method.

## Discussion

The formation of a solid tumor is a multi-staged process that results in abnormal gene expression. Epigenomic changes are an important cause of tumorigenesis, and methylated cytosine has been previously reported as an important factor in this process (20,21). During the process of proliferation, cellular nucleoside production is increased. Modified intracellular nucleosides are difficult to degrade, and can only be excreted through the urine. The level of urinary modified nucleosides can therefore be used as a reflection of the *in vivo* metabolic rate of nucleosides. During the process of canceration, the level of urinary modified nucleosides markedly increase, therefore, analysis of modified nucleosides in the urine could facilitate preliminary tumor detection. DNA methylation is one of the predominant mechanisms leading to loss of gene function (22). Data has shown that CRC is a tumor type that is prone to high levels of abnormal gene methylation (23,24). Previous studies have indicated that methylation of multiple genes can accurately reflect CRC much more than single gene methylation (25,26).

Previous reports have shown that DNA fragments from tumor genes have been successfully isolated from the urine of patients who have been diagnosed with CRC (27,28). These studies have therefore demonstrated that DNA testing from urine samples may be a feasible method for the diagnosis of early-stage cancer. As compared with plasma or sera diagnoses, a urinalysis can eliminate the possibility of blood infection (29). In addition, the amount of tumor DNA that can be obtained from peripheral blood serum is low (ng), as compared with tissue samples, and the majority of DNA can be readily degraded during the process of modification by NaHSO_3_. For trace DNA samples, the sensitivity of the conventional n-MSP method requires improvements (18).

Ahlquist *et al* (30) found that DNA isolated from feces has improved sensitivity in methylated gene detection as compared with DNA extracted from peripheral blood (30,31), particularly in early-stage CRC. This may be associated with the direct contact with the colorectal tissue and the alkaline environment in which colorectal cells are exposed to. Conversely, low DNA levels in the peripheral blood and the presence of numerous factors in the plasma could influence the PCR reaction (32,33). In the present study, the methylated NDRG4 gene expression rates in the tumor and adjacent tissues, were 81 and 8.3%. This rate of methylation at the CpG site, however, was not significantly associated with age, differentiation level or TNM stage.

The NDRG4 transcript is 32 Kb long, consisting of 17 exons and 16 introns. The function of NDRG4 in tumor development remains unclear, but numerous studies have indicated that NDRG4 is associated with the growth, differentiation and metastasis of the tumor (17,34,35). The 5′ regulatory region of NDRG4 contains the CpG island, and is often methylated in the occurrence and development of CRC, thus the methylation of NDRG4 is considered to be an important biological feature of colorectal cancer(17). Melotte *et al* (16) identified that NDRG4 is a biomarker candidate tumor suppressor gene in CRC, and methylation of the NDRG4 promoter can be used as a biological marker for the detection of colorectal cancer. The sensitivity and specificity of NDRG4 methylation were 61 and 93% in the fecal samples analyzed.

In the present study, n-MSP was used to detect methylated NDRG4. The sensitivity and specificity was 76.2 and 89.15% in stool samples and 72.6 and 85% in urine samples. This indicated that, as compared with blood samples, methylated NDRG4 in DNA isolated from feces and urine, can be used as a non-invasive biomarker for the detection of early stage CRC. This method could be used to enrich the current diagnostic methods of CRC. Urine contains significantly less protein than that of blood, therefore the isolation of DNA fragments from urine is facilitated. Urine samples are easy to obtain, and have high stability, reproducibility and specificity. The methylation of NDRG4 may therefore become a standard measure in the clinical diagnosis of CRC.

In conclusion, the present study has demonstrated that the detection of NDRG4 methylation status in feces and urine has sufficient sensitivity and specificity as a diagnostic measure in CRC. Considering the advantages of easy acquisition of urine samples, detection of NDRG4 methylation in urine could be suitable for detecting low levels of methylated genes, to facilitate the early diagnosis of CRC.

## Figures and Tables

**Table I:** Primer sequences, annealing temperatures, and product sizes used for nested methylation-specific polymerase chain reaction, to analyze the methylation status of the NDRG4 promoter.

Primer sequence	Temperature, °C	Product size, bp
Outer		
NDRG4-F: 5′-GGTTYGTTYGGGATTAGTTTTAGG-3′ NDRG4-R: 5′-CRAACAACCAAAAACCCCTC-3′	56	257
Inner		
Methylated		
NDRG4-UF: 5′-GATTAGTTTTAGGTTTGGTATTGTTTTGT-3′ NDRG4-UR: 5′-AAAACCAAACTAAAAACAATACACCA-3′	66	100
Un-methylated		
NDRG4-MF: 5′-TTTAGGTTCGGTATCGTTTCGC-3′ NDRG4-MR: 5′-CGAACTAAAAACGATACGCCG-3′	66	88

bp, base pairs; F, forward; R, reverse; U, unmethylated; M, methylated.

**Table II:** Association between methylated NDRG4 gene expression and clinicopathological parameters of colorectal cancer.

		Tissue		Stool		Urine		Blood	
					
Object	Total	Methylated	Unmethylated	Methylation	Unmethylation	Methylated	Unmethylated	Methylated	Unmethylated
Age, years									
<65	36	29	7	26	10	25	11	19	17
≥65	48	39	9	38	10	36	12	27	21
Differentiation									
High-middle	57	49	8	45	12	44	13	29	28
Low	27	19	8	19	8	17	10	17	10
Lymph node metastasis									
Exist	55	50	5^a^	48	7^a^	42	13	30	25
None	29	18	11	16	13	19	10	16	13
TNM stage									
I–II	48	42	6	39	9	34	14	26	22
III–IV	36	26	10	25	11	27	9	20	16

a P<0.05.

TNM, TNM Classification of Malignant Tumors cancer staging system (36).
